# Bull's eye or typhoon eye? Psychological distress and associated factors in Wuhan and surrounding areas during the COVID-19 pandemic

**DOI:** 10.1017/gmh.2022.18

**Published:** 2022-03-11

**Authors:** Haiyan Gao, Xi Chen, Yuchun Zou

**Affiliations:** 1 The faculty of Humanities and Social Sciences, Beijing University of Technology, Beijing, China; 2 The Jockey Club School of Public Health and Primary Care, The Chinese University of Hong Kong, Hong Kong, China; 3 Institute of Sociology, Chinese Academy of Social Sciences, Beijing, China

**Keywords:** China, COVID-19 pandemic, information-seeking behavior, perceived discrimination, psychological distress, social assistance

## Abstract

The COVID-19 pandemic caused significant psychological consequences among the public, especially for people in the epicenter. This study examined the ‘bull's eye’ model by comparing the level of psychological distress and the effect of different stressors in Wuhan (the original epicenter) with that in the surrounding areas in Hubei Province during the pandemic. Data were obtained from a cross-national survey of 10 478 respondents between the ages of 18 and 80 years in Hubei Province during the peak of the pandemic. Results of the ordinary least squares regression models showed that Wuhan residents experienced more psychological distress than those in the surrounding areas. Social and economic problems caused by the pandemic, risk exposure, perceived discrimination, and information-seeking behaviors were positively associated with distress. Social assistance was negatively associated with distress. Findings were consistent with the bull's eye model by revealing both a higher level of psychological distress and a stronger effect of stressors among the Wuhan residents than with those in low-risk areas. Thus, policymakers and psychological workers should provide adequate psychological services in high-risk areas. Lowering risk exposure, reducing discrimination against people in the epicenter, and improving information quality are essential to alleviate their psychological distress.

## Introduction

The 2019 Coronavirus disease (COVID-19) first reported in Wuhan, China, in December 2019, has spread rapidly worldwide. More than 426.6 million infected cases of COVID-19 and nearly 5.9 million related deaths (World Health Organization, 2022) have been reported as of 20 February 2022. It has impacted the lifestyles, economy, and the physical and mental health of individuals worldwide (Wang *et al*., 2021*a*, 2021*b*). Governments have taken different measures, including containment-related actions and economic and health policies. Some of these measures are beneficial for the wellbeing of the population (Lee *et al*., 2021), whereas some measures such as lockdown, may cause mental health problems, including anxiety and depression (Le *et al*., 2020; Tran *et al*., 2020). In addition, COVID-19 survivors were reported to experience symptoms of depression and posttraumatic stress disorder (Hao *et al*., 2020; Renaud-Charest *et al*., 2021). Examining and exploring individual's mental health and its associated factors are crucial for both academic researchers and public health policymakers.

Various risk and protective factors may be associated with psychological wellbeing during the pandemic (Drapeau *et al*., 2012). Existing COVID-19 studies have identified a number of risk factors, such as COVID-19 exposure, financial stress, home confinement, and social discrimination. By contrast, protective factors may include higher income, higher education, more knowledge about COVID-19, and material and psychological support (Mazza *et al*., 2020; Rajkumar, 2020; Wang *et al*., 2020*a*, 2020*b*; Browning *et al*., 2021; Qin *et al*., 2021). Among them, the relationship between distance to the epicenter and mental health drew considerable attention among scholars. Some studies showed that individuals living in disaster hotspots may experience a heightened state of anxiety, psychological distress, and fear than those living in the surrounding areas (Fischhoff *et al*., 2003; Marshall *et al*., 2007; Wu *et al*., 2009). This phenomenon is known as the ‘ripple effect’ or ‘bull's eye’ model. In the case of COVID-19, the bull's eye model was observed in China (Qiu *et al*., 2020), Italy (Liang *et al*., 2020), India (Agarwal, 2020), Peru (Yáñez *et al*., 2020), and Africa (Coker *et al*., 2020). By contrast, the ‘psychological typhoon eye’ effect, which refers to the phenomenon that the closer to the center of an epidemic, the less severe the mental health problems, was also observed in previous epidemics (Xie *et al*., 2011); the current COVID-19 pandemic (Xu *et al*., 2020; Zhang *et al*., 2020); and other forms of disasters such as terrorist attacks (Schlenger *et al*., 2002; Li *et al*., 2020), earthquake (Jia *et al*., 2008; Li *et al*., 2009), and exposure to contaminating substances (radioactivity, chemical pollution, etc.) (Maderthaner *et al*., 1978; Zheng *et al*., 2015). The contradictory findings on the relationship between distance to epicenter and psychological wellbeing may result from different targeted groups, different measures of psychological wellbeing, and measurement perspectives (actor or bystander) (Wen *et al*., 2020). This study aims to investigate the differences in psychological wellbeing between people in the epicenter of the pandemic and those in the surrounding areas. If people in the epicenter (Wuhan) suffered more from psychological distress and pandemic-related stressors, the findings support the bull's eye model. Otherwise, the findings are consistent with the typhoon eye model. In addition, our study advances past literature by examining how risk and protective factors may have different effects on the psychological wellbeing between people in the epicenter and surroundings. In other words, people in the epicenter and surrounding areas may not only experience different levels of stressors (i.e. differential exposure) but are also different in their vulnerability to these stressors (i.e. differential effect).

Despite the numerous surveys conducted, Hubei residents are surprisingly underrepresented in previous surveys (Gao *et al*., 2020). Our study, with a large sample of Wuhan and surrounding area residents, examines whether stressors and resources function differently for populations in the epicenter and those in low-risk areas. The comparisons will contribute to a better understanding of COVID-19-fueled psychological wellbeing study and the debate on typhoon eye and bull's eye effects.

## Methods

### Participants

This study obtained data from the ‘Public Attitude toward the COVID-19 Pandemic in Hubei Province’ survey launched by The China Academy of Science and Technology Development Strategy, the Social Policy Research Institute at Renmin University and the Institute of Sociology of the Chinese Academy of Social Sciences. The survey was conducted between 4 and 8 February 2020, approximately 2 weeks after Wuhan announced its closure on 23 January 2020. The quarantine order was then imposed on 15 other cities in Hubei.

**Fig. 1.**
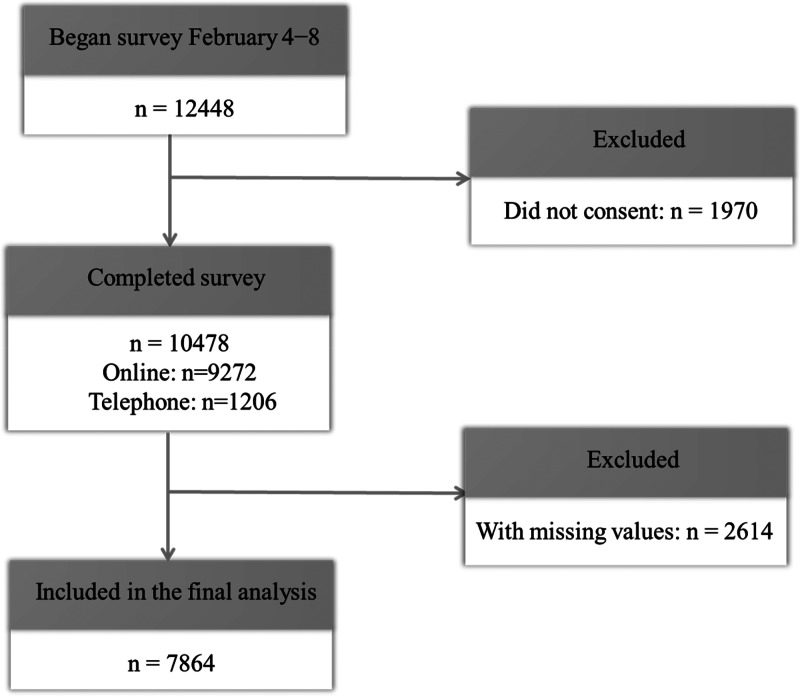
Study flow diagram.

The survey targeted all residents aged between 18 and 80 in the urban and rural areas of Hubei. A web-based survey and a telephone survey were employed. The online survey was carried out on Epanel, a professional survey platform in China. The telephone survey aimed to recruit non-Internet users who were typically older, living in rural areas, and with low socioeconomic status. Trained research assistants conducted the telephone survey and used a snowball sampling. This study sought the review and approval by The Chinese Academy of Social Science Ethics Committee before it began. It also assured the anonymity and confidentiality of all the participants. A total number of 9272 participants completed the online survey, and 1206 participants attended the telephone survey. After deleting cases with missing values, the final sample was 7864. Given that the amount of missing value is large (25%), we also used multiple imputation methods to impute the missing observations and conducted the same analysis. The difference in the results was insignificant from the sample using the complete responses. Thus, we reported these findings using the complete information. Figure 1 showed a diagram of the study flow.

### Measures

#### Psychological distress

Psychological distress was measured by asking the participants to rate on a five-point Likert scale how anxious, fearful, and worrisome they felt about the COVID-19 pandemic (1 = not at all to 5 = extremely). This simple instrument was adapted from Wang and Ying's (2020) study of psychological wellbeing among the public during the COVID-19 pandemic in China. The average score of the three items was used to represent the level of psychological distress, with higher scores indicating higher levels of distress (three-item scale; *α* = 0.82).

#### COVID-19-related stressors

COVID-19-related stressors are categorized into four groups: exposure to COVID-19, the influence on daily life and work, information-seeking behavior, and perceived discrimination. For the measurement of risk exposure, participants were asked whether they and/or any of their family members, neighbors, and residents in the same neighborhood had developed a fever recently (the most common symptom of COVID-19) (yes *v.* no). High-risk exposure was identified if the respondent answered yes to any of these questions. For the measurement of influence on daily life and work, participants were asked to identify the three stressors they were most concerned about during the pandemic out of a list of stressors, such as ‘afraid to go to the hospital’, ‘reduced income’, and ‘inconvenient daily life’. Considering the prevalence of the stressors included in the list during the pandemic, asking the participants to identify the top three ones can differentiate the significance of these stressors. The selected items were coded as ‘1’; otherwise, ‘0’.

Information-seeking behaviors were measured by attention on COVID-19 information and perceived source credibility. Attention on COVID-19 information was gauged by the level of attention the participants paid to six different information contents about COVID-19, such as statistics of infection, local necessity supplies, and government responses to the pandemic (1 = paid no attention at all to 4 = paid a lot of attention). The mean score of the six items was used to represent the level of attention on information regarding COVID-19 (six-item scale, *α* = 0.88). As for perceived source credibility, the participants' views were assessed on how reliable for them the COVID-19 information was as provided by the following sources: central government officials, local government officials, community/village officials, medical experts/scientists, relatives and friends, central media, local media, and social media (1 = completely unreliable to 4 = completely reliable). Two factors were obtained with factor analysis. They were named perceived reliability of official information and perceived reliability of unofficial information.

Perceived discrimination was measured by asking the participants ‘whether you felt being discriminated due to the pandemic’ (1 = not at all to 4 = felt strongly). Previous studies have used a similar single item to assess perceived discrimination because of COVID-19 (Xin *et al*., 2020).

#### Social assistance

The measure of social assistance was adapted from the Received Social Support Questionnaire (Kaniasty and Norris, 1995), which focuses on both emotional support (e.g. expressions of assurance) and tangible assistance (e.g. receiving food) in the wake of traumatic events. Social assistance was assessed by asking the participants whether they received assistance from friends/relatives or neighborhoods through the provision of masks/drugs, groceries, child/elderly care, and expressions of assurance during the pandemic. Responses for each item were recoded as 0 for never and 1 for having received.

#### Control variables

Sociodemographic factors such as age, gender, education, monthly income, subjective social status, party membership, and occupations are included as control variables. Table 1 shows the measurement of these variables.

**Table 1.** Descriptive statistics

Variable	Description	Median/%	IQR
Sociodemographic			
Low-risk/high-risk area	1 = Wuhan area	14.2%	–
	0 = non-Wuhan	85.8%	–
Gender	1 = male	50.4%	–
	0 = female	49.6%	–
Communist party membership	1 = CCP	15.2%	–
	0 = non-CCP	84.8%	–
Area	1 = urban	80.4%	–
	0 = rural	19.6%	–
Education	1 = junior or below	20.3%	–
	2 = high school	24.0%	–
	3 = college or above	55.7%	–
Monthly income	1 = no income	7.90%	–
	2 = RMB 2000 or below	18.3%	–
	3 = RMB 2000–4000	35.1%	–
	4 = RMB 4001–6000	21.6%	–
	5 = RMB 6001–8000	10.1%	–
	6 = RMB 8000 or above	7.0%	–
Occupation	1 = farmers/blue-collar workers	17.0%	–
	2 = private businessmen/the service workers	16.9%	–
	3 = white-collar workers	37.0%	–
Age	in years	31	20
Subjective social status	1 = lower class; 4 = middle-upper class	3	1
Dependent variables			
Psychological distress	1 = least distressed; 5 = most distressed	4.33	1
Epidemic-related stressors			
Unpredictable course of the pandemic	1 = yes; 0 = no	41.8%	
Being infected	1 = yes; 0 = no	36.8%	–
Being afraid to go to the hospital	1 = yes; 0 = no	56.4%	–
Reduced income	1 = yes; 0 = no	16.9%	–
Work/business was affected	1 = yes; 0 = no	24.5%	–
Inconvenient daily life	1 = yes; 0 = no	30.0%	–
Unstable social environment	1 = yes; 0 = no	41.1%	–
Economic stagnation	1 = yes; 0 = no	34.1%	–
Exposure to persons with fever	1 = yes; 0 = no	45.2%	–
Perceived discrimination	1 = not at all; 4 = feeling strongly	1	1
Attention on COVID-19 information	1 = not at all; 4 = very much	3.67	0.56
Perceived reliability of official information	1 = not trustworthy; 4 = completely trustworthy	3.33	0.67
Perceived reliability of unofficial information	1 = not trustworthy; 4 = completely trustworthy	3	0.5
Social assistance			
Medical supplies	1 = yes; 0 = no	31.8%	–
Daily supplies	1 = yes; 0 = no	21.6%	–
Caring for children	1 = yes; 0 = no	18.6%	–
Communication and assurance	1 = yes; 0 = no	34.1%	–

### Data analysis

Descriptive statistics were reported as mean and standard deviation (s.d.) for normally distributed continuous variables or median and interquartile range (IQR) in the case of skewed distributions (Table 1). Normality of distribution of statistics was tested by Skewness/Kurtosis tests for normality. For the regression analysis, we first used ordinary least squares (OLS) regression to examine the effects of sociodemographic variables on psychological distress. Predictors significant at univariable association (*p* < 0.1) were included in the following multivariable regression models. OLS regressions with robust standard errors were carried out to examine the effects of COVID-19-related stressors and social assistance on psychological distress after adjusting for sociodemographic variables. Lastly, two-way interactions between high- *v.* low-risk areas and each risk and protective factor were computed to examine whether these relationships vary by local severity of the COVID-19 pandemic. All the analyses were performed using Stata 16.0. Unstandardized coefficients with 95% confidence intervals were reported. A *p* value of 0.05 was set as the level of statistical significance.

## Results

Table 2 shows the associations between sociodemographic variables and psychological distress among Hubei residents. Age showed a nonlinear relationship with psychological distress. Although a positive relationship existed between age and distress below age 30, age was negatively related to distress over 30. Female participants were more likely to be distressed than males during the pandemic. Members of the Communist Party of China (CCP) were more likely to experience distress than non-CCP members partly because they were pushed to the front line of the community battle against COVID-19 and experienced high levels of risk exposure to COVID-19. Other indicators of socioeconomic status, including education, income, occupation, and subjective social status, were not associated with psychological distress. The buffering effect of individual's socioeconomic factors did not show up during the peak of COVID-19.

**Table 2.** Association between sociodemographic variables and psychological distress

	Coefficient	s.e.
Age	0.013**	0.004
Age^2^	−0.0002***	0.00005
Gender (ref: female)		
Male	−0.05**	0.017
CCP membership (ref: non-CCP member)		
CCP member	0.068**	0.024
Area (ref: rural)		
Urban	−0.003	0.023
Education (ref: middle school or below)		
High school	−0.038	0.027
College or above	−0.026	0.027
Monthly income (ref: no income)		
⩽2000	−0.034	0.064
2001–4000	−0.012	0.063
4001–6000	−0.017	0.064
6001–8000	0.009	0.067
>8000	0.001	0.069
Occupation (ref: peasant/blue-collar worker)		
Service worker/individual business owner	0.038	0.024
White-collar worker	−0.012	0.021
Subjective social status	0.005	0.010
Constant	4.099***	0.093

* *p* < 0.05; **p < 0.01; ***p < 0.001.

Table 3 shows the effects of COVID-19-related stressors and social assistance on psychological distress. The results in Model 1 show that Wuhan residents experienced more psychological distress than residents in other areas of Hubei (*β* = 0.078, *p* < 0.01), which showed support for the bull's eye model during the COVID-19 pandemic in Hubei.

**Table 3.** Factors associated with psychological distress among residents in Hubei Province

	Model 1	Model 2	Model 3	Model 4
Local severity of infection (ref: non-Wuhan area)				
Wuhan	0.078** (0.024)	0.011 (0.021)	0.023 (0.035)	0.023 (0.031)
COVID-19-related stressor				
Unpredictable course of the pandemic		0.089*** (0.016)		0.057* (0.024)
Being infected		0.045** (0.016)		0.024 (0.023)
Traffic affected		−0.061*** (0.017)		−0.070** (0.025)
Unstable social environment		0.064*** (0.016)		0.072** (0.024)
Economic stagnation		0.076*** (0.016)		0.074** (0.024)
Perceived discrimination		0.128*** (0.007)		0.129*** (0.01)
Attention on COVID-19 information		0.430*** (0.021)		0.445*** (0.032)
Perceived reliability of official information		0.060** (0.019)		0.073** (0.027)
Perceived reliability of unofficial information		0.063*** (0.012)		0.059*** (0.018)
Social assistance				
Receiving medical supply from friends or neighborhood			−0.057* (0.026)	−0.033 (0.022)
Constant	4.059*** (0.077)	2.186*** (0.113)	4.113*** (0.140)	2.206*** (0.167)
Adjusted *R*^2^	0.008	0.160	0.008	0.154

All models adjusted for sociodemographic variables, including age, gender, education, occupation, monthly income, CCP membership, rural/urban residence, and subjective social status. Only significant variables were shown in the table.

Standard errors are shown in parentheses.

* *p* < 0.05; **p < 0.01; ***p < 0.001.

As shown in Model 2, several COVID-19-related stressors were positively associated with psychological distress, including the unpredictable course of the pandemic (*β* = 0.089, *p* < 0.001); afraid of being infected (*β* = 0.045, *p* < 0.01); traffic affected (*β* = −0.061, *p* < 0.001); unstable social environment (*β* = 0.064, *p* < 0.001); economic stagnation (*β* = 0.076, *p* < 0.01); perceived discrimination (*β* = 0.175, *p* < 0.001); attention on COVID-19 information (*β* = 0.430, *p* < 0.001); perceived reliability of official information (*β* = 0.060, *p* < 0.01); and perceived reliability of unofficial information (*β* = 0.063, *p* < 0.001).

Model 3 examines the effects of different types of social assistance on psychological wellbeing. Social assistance with medical supplies was associated with less psychological distress (*β* = −0.057, *p* *<* 0.05). Social assistance with daily necessities, child/elderly care, and expressions of assurance was not significantly associated with psychological distress.

**Fig. 2.**
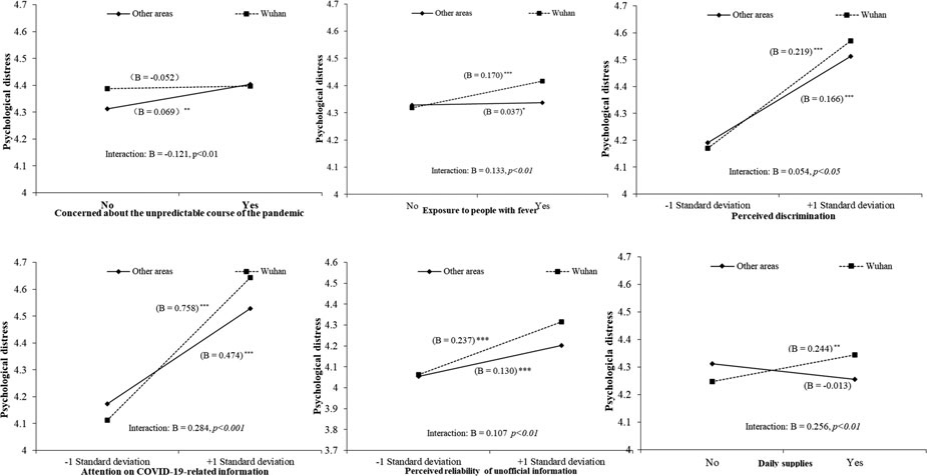
Simple slopes (unstandardized coefficient). *p < 0.05; **p < 0.01; ***p < 0.001.

We then computed the interaction terms between various stressors and social assistance with Wuhan/non-Wuhan area. Table 4 presents the significant interactions. Figure 2 shows the results of simple slope analyses. As shown in Fig. 2, the effects of exposure to higher risk of COVID-19, perceived discrimination, attention on COVID-19-related information, perceived reliability of unofficial information, and social assistance on daily necessities on psychological distress were stronger among Wuhan (i.e. epicenter) residents. In other words, Wuhan residents were more vulnerable to these stressors than their non-Wuhan counterparts. By contrast, the relationship between the concern about the unpredictable course of the pandemic and distress was stronger among people in the surrounding areas of the epicenter. This phenomenon might be because Wuhan residents could no longer expect a worse situation, whereas other areas were still in danger of worsening.

**Table 4.** Interactions between risk and protective factors and high/low-risk areas on psychological distress

	Model 1	Model 2	Model 3	Model 4	Model 5	Model 6
High-risk area (Wuhan)						
×Unpredictable course of the pandemic	−0.121** (0.042)					
×Risk exposure to COVID-19		0.133** (0.048)				
×Perceived discrimination			0.054* (0.021)			
×Attention on COVID-19 information				0.284*** (0.064)		
×Perceived reliability of unofficial information					0.107** (0.039)	
×Receiving daily necessities from friends or neighborhoods						0.256** (0.139)
Constant	4.044*** (0.080)	4.081*** (0.094)	3.891*** (0.095)	2.601*** (0.115)	3.775*** (0.106)	2.650*** (0.087)

All models adjusted for sociodemographic variables, including age, gender, education, occupation, monthly income, CCP membership, rural/urban residence, and subjective social status. The main effects of the variables were included in the analysis, but only the significant interaction terms were reported.

Standard errors are shown in parentheses.

*p < 0.05; **p < 0.01; ***p < 0.001.

## Discussion

Our study contributes to research on the psychological consequences of the COVID-19 pandemic by analyzing the different effects of stressors and resources on psychological wellbeing between a hotspot and less afflicted areas during the COVID-19 pandemic. Varying degrees of severity and hazards of the pandemic differ in the center, and other areas may also show differences in different people's psychological distress levels. This finding conforms to the bull's eye model by revealing that Wuhan (the epicenter) residents experience higher levels of psychological distress than those in the surrounding low-risk areas. Furthermore, our analysis showed that certain stressors, such as risk exposure, information-seeking behaviors, and perceived discrimination, had a stronger effect on the psychological wellbeing of populations in the hotspot than people in surrounding areas. It can be interpreted as another form of bull's eye effect. Unexpectedly, worrying about losing control of the pandemic had a larger impact on the psychological distress of residents in surrounding areas than that of residents in the hotspot (Wuhan). During the peak of the pandemic, Wuhan experienced a dire situation, as the number of confirmed cases increased by thousands each day, and medical systems were overloaded (Bloomberg, 2020). Thus, Wuhan residents may not expect a situation worse than they were already experiencing at that time. However, the concern that a similar situation could occur in their respective cities or villages was high among the residents of surrounding areas.

Although our findings seemed to be consistent with the bull's eye model, other studies are warranted to further explore under which condition the bull's eye and typhoon eye models may apply. A wide range of literature on different forms of disasters was reviewed, including geographically circumscribed acute events such as an earthquake/a terrorist attack and chronic diffusion such as radioactivity/chemical pollution. Generally, for chronic diffusion events such as nuclear power stations (Maderthaner *et al*., 1978) and lead-zinc mining (Zheng *et al*., 2015), the typhoon eye effect was more likely to be reported due to fear of contagion. However, as for the acute rate of diffusion events such as an earthquake or a terrorist attack, empirical evidence showed mixed results (Jia *et al*., 2008; Li *et al*., 2009). COVID-19 has the features of high infectivity and acute rate diffusion. Studies also showed mixed results. Some studies are supportive of the typhoon eye effect (Yang *et al*., 2020; Wang *et al*., 2020*a*, 2020*b*; Zhang *et al*., 2021), while some are consistent with the bull's eye effect (Huang *et al*., 2020; Liang *et al*., 2020; Lateef *et al*., 2021). The results of our research that the generalized distress and stressors among residents in Wuhan were higher than the surrounding areas indicated a bull's eye model during the peak of the outbreak. Future studies with strict research design on samples, measurements, distance, and stages should be carried out to examine the specific conditions for the bull's eye effect and typhoon eye effect.

In our study, results on socioeconomic demographics showed that being female, middle-age, and members of CCP endured increased levels of psychological distress during the peak of the epidemic. Resource-related factors such as education, income, prestige of occupation, and subjective social status did not show significant effect on psychological distress. The total explanative power was very small at the stage. However, later study showed that as the pandemic subsided, the psychological wellbeing of people of all educational levels rebounded; nonetheless, the recovery was greater and faster for those with tertiary education (Jin *et al*., 2022). Studies on the waning and quarantine period also showed that people with better family economic status had fewer symptoms of mental problems (Browning *et al*., 2021; Li *et al*., 2021). Our results might suggest that during the peak of the outbreak, people of all social classes experienced a similar level of uncertainty, anxiety, and distress. The socioeconomic inequality on mental health may not be as significant as in the other stages of the pandemic.

Information-seeking behaviors were categorized as stressor factor, and our result actually showed that attention paid to COVID-19-related information was significantly associated with psychological distress. It suggests that excessive intake of information may be harmful to individuals' psychological wellbeing. In addition, perceived reliability of official and unofficial information was positively associated with psychological distress. Given the sensationalized news headlines and images regarding the rapid spread of the virus reported by different media during the peak of the pandemic in China, people who perceive the information as more trustworthy were likely to suffer more psychological distress. Moreover, the relationship between perceived reliability of COVID-19 information from informal sources (e.g. friends and relatives) and psychological distress was more salient among people in the epicenter. It may be because the information from informal sources often contains misinformation and disinformation that may induce stress and anxiety (Goodwin *et al*., 2009; Neria and Sullivan, 2011; Xiong *et al*., 2020). Compared with populations in low-risk areas, residents in the epicenter who perceived COVID-19 information from informal sources as valid may experience higher levels of confusion and become pessimistic about getting the pandemic under control and thus expressed more psychological distress.

Inconsistent with other COVID-19 studies (e.g. Duan *et al*., 2020), the beneficial effects of material and psychological support on psychological wellbeing were limited in our study. Only social assistance with medical supplies exerted a significant effect on psychological distress. A possible explanation for this finding is that the most serious stressors during the peak of the outbreak are related to the risk of infection, financial loss, and unstable social environment. Control over the pandemic and recovery to normal life are the most urgent and possibly most effective solutions to alleviate psychological distress. Although the beneficial role of social support in psychological wellbeing was limited during the peak of the pandemic, social support may play a considerable role in reducing distress in other stages of the pandemic (Li *et al*., 2021; Szkody *et al*., 2021).

Despite these significant findings, the current study was not without limitations. First, this research was a cross-sectional study conducted during the peak of the outbreak. Thus, conclusions about the bull's eye model and predictors of psychological distress were confined to this stage. Longitudinal studies tracking the psychological wellbeing change from the initial peak to 1-month-later waning period showed that a descending trend of mental problems was observed; however, post-traumatic stress disorder (PDST) symptoms persisted (Wang *et al*., 2020*a*, 2020*b*). Studies can further explore the difference in PDST symptoms between low- and high-risk areas. Second, despite its large sample size, the survey was mainly conducted online. Although additional telephone interviews were carried out to include people with limited access to computers and cellphones, sample biases remained. Third, several measures, such as psychological distress and social assistance, were not validated among the Chinese population and may result in measurement errors. However, such measures were adapted from previous studies, and internal validity was satisfactory. Future studies may further test the validity of the variables among the Chinese population. Finally, this study did not study suicide, one extreme consequence of COVID-19. Research has shown mixed findings about the effect of COVID-19 on suicide rate (McIntyre *et al*., 2021; Lee *et al*., 2022). Thus, further studies can investigate the suicide rate trend among different risk areas.

The findings showed a higher level of psychological distress among Wuhan residents than those in low-risk areas during the peak of the pandemic, which is consistent with the bull's eye model. The findings also reveal the similarities and differences in the effect of stressors and resources on psychological wellbeing between the hotspot and surrounding areas. While some stressors functioned similarly for the residents of Wuhan and surrounding areas, other stressors, such as information-seeking behaviors, perceived discrimination, and risk exposure, may lead to worse mental health among the residents in the epicenter. Based on these findings, policymakers and psychological workers should provide adequate psychological services to residents in high-risk areas. Previous studies showed that cognitive behavioral therapy (Ho *et al*., 2020) as well as its new form –Internet cognitive behavioral therapy (Zhang and Ho, 2017), a treatment that can be delivered via the Internet, has been proven to be effective in the treatment of psychiatric symptoms such as insomnia (Soh *et al*., 2020). These treatments can be further promoted to improve the accessibility and availability of mental health service for patients worldwide.
